# DNA damage response- and JAK-dependent regulation of PD-L1 expression in head and neck squamous cell carcinoma (HNSCC) cells exposed to 5-fluorouracil (5-FU)

**DOI:** 10.1016/j.tranon.2021.101110

**Published:** 2021-05-02

**Authors:** Claire Lailler, Michele Lamuraglia, Floriane Racine, Christophe Louandre, Corinne Godin, Bruno Chauffert, Antoine Galmiche, Zuzana Saidak

**Affiliations:** a Laboratoire de Biochimie, Centre de Biologie Humaine (CBH), CHU Sud, Amiens, France; b UR7516 “CHIMERE”, Université de Picardie Jules Verne, Amiens, France; c Laboratoire d'Imagerie Biomédicale (LIB), Sorbonne Université, CNRS, INSERM, Oncologie Médicale, CHU Sud, Amiens, France

**Keywords:** DEG, differentially expressed genes, EGFR, Epidermal Growth Factor Receptor, FDR, false discovery rate, GO, Gene Ontology, HNSCC, head and neck squamous cell carcinoma, ICB, immune checkpoint blockers, IFN-γ, Interferon-γ, NGF, Nerve Growth Factor, PD-L1, Programmed cell Death 1-Ligand 1, R/M, Recurrent/Metastatic, TS, Thymidylate synthase

## Abstract

**Objectives:**

The immune checkpoint molecule PD-L1 (CD274) is a crucial regulator of the tumor immune response. Its expression has been reported in the therapeutic context in Head and Neck Squamous Cell Carcinoma (HNSCC), but it remains unclear how therapeutically approved molecules regulate PD-L1 expression in HNSCC cells.

**Materials and methods:**

Three HNSCC cell lines (BICR6, PE/CA-PJ34 and PE/CA-PJ41) were used to analyze PD-L1 expression by immunoblotting, immunofluorescence and QPCR. Freely-available single cell RNAseq data from HNSCC were also used.

**Results:**

5-Fluorouracil (5-FU) increased the expression of PD-L1 with high efficacy in HNSCC cells. Single cell RNAseq data suggested the specificity of the regulation of PD-L1 in this context. The effect of 5-FU on PD-L1 expression was related to its genotoxic effect and was prevented by extracellular application of thymidine or using a chemical inhibitor of the DNA damage Response kinases ATM/ATR. We found that the effect of 5-FU was additive or synergistic with IFN-γ, the canonical inducer of PD-L1 in epithelial cells. QPCR analysis confirmed this finding and identified JAK-dependent transcriptional activation of PD-L1/CD274 as the underlying mechanism. The induction of PD-L1 by 5-FU was partially prevented by Epidermal Growth Factor Receptor (EGFR) inhibition with cetuximab.

**Conclusion:**

Our study highlights the specific DNA Damage Response- and JAK- dependent induction of PD-L1 by 5-FU in HNSCC cells. This induction is regulated by the cytokine context and is potentially therapeutically actionable.

## Highlights

• PD-L1 is an important immune checkpoint molecule expressed by HNSCC.
• 5-FU induces PD-L1 expression in HNSCC cells.
• PD-L1 upregulation is DNA damage Response- and JAK-dependent.
• 5-FU potentiates the effect of the inflammatory cytokine Ifn-γ.
• Targeting EGFR with cetuximab blunts PD-L1 expression induced by 5-FU.

## Introduction

Head and Neck Squamous Cell Carcinoma (HNSCC) are a heterogeneous group of tumors that require multimodal treatment with surgery and adjuvant radio(chemo)therapy [1,2]. Despite adapted initial treatments, local recurrence and metastasis remain frequent and constitute an indication for chemotherapy and immunotherapy [1,2]. The recent introduction of immune checkpoint blockers (ICB) has challenged the medical practice for advanced stages of HNSCC. Nivolumab and pembrolizumab, two monoclonal antibodies targeting the interaction between the molecule PD-L1 (Programmed cell Death 1-Ligand 1, encoded by the gene *CD274*) and its receptor PD1, are approved for the treatment of recurrent/metastatic (R/M) HNSCC [3,4]. PD1 targeting used alone is considered effective in almost 20% of R/M HNSCC patients [3,4]. PD-L1 expression is usually analyzed by immunohistochemistry and scored using the CPS (Combined Positive Score), defined as the sum of PD-L1-positive cancer cells and monocytes/lymphocytes divided by the total number of tumor cells x 100 [5]. The use of CPS reflects the dual expression of PD-L1 in cancer and tumor-infiltrating immune cells. In addition to the interest of CPS in predicting the benefit of ICB, PD-L1 can be a biomarker of negative or positive prognostic value, depending on its expression on epithelial or immune cells, respectively [6,7].

Chemotherapy is currently indicated for patients with advanced HNSCC, *i.e.* those directly presenting with R/M HNSCC or those with high risk tumors (nodal extracapsular spread or invaded surgical margins). Platinum salts (cisplatin or carboplatin), taxanes (docetaxel or paclitaxel), and 5-fluorouracil (5-FU) are used in this setting [8]. After transformation into FdUMP, 5-FU mainly interferes with nucleic acid metabolism by blocking the enzyme thymidylate synthase (TS) and inhibiting *de novo* pyrimidine synthesis. This effect of 5-FU results in an inhibition of DNA synthesis and a block in cell cycle progression [9]. A homeostatic reaction called the DNA Damage Response (DDR) is induced in this context and constitutes a determinant of HNSCC sensitivity to 5-FU [9]. Cetuximab, a targeted therapy directed against the Epidermal Growth Factor Receptor (EGFR) counteracts oncogenic signaling downstream of EGFR in HNSCC cells [2].

An immune response directed against cancer cells is emerging as a mechanism that contributes to the efficacy of therapeutic protocols used against solid tumors [10,11]. Interestingly, a recent study examining HNSCC resected after neoadjuvant chemotherapy reported increased PD-L1 expression in cancer cells in this context [12]. In 71% of tumor samples from patients that received induction chemotherapy with TPF (docetaxel + platinum + 5-FU), increased levels of PD-L1 and a significant increase in the density of CD8+ *T* cell infiltrate were detected [12]. Previous *in vitro* studies found that cisplatin induced PD-L1 expression in HNSCC cells [13,14]. It is unclear which chemotherapeutic drug is the most effective at increasing PD-L1 expression in HNSCC cells and how PD-L1 expression is regulated in this context.

## Materials and methods

**Cell culture**. The cell lines BICR6, PE/CA-PJ34 and PE/CA/PJ-41 are described in detail in the supplementary Materials and Methods section. Cells were cultured in Dulbecco's Modified Eagle's Medium (DMEM) supplemented with 10% fetal calf serum, 2 mM glutamine, and penicillin / streptomycin.

**Reagents and chemicals.** All chemical reagents were purchased from Sigma, unless stated otherwise. Afatinib and VE821 were purchased from Selleckchem. The JAK inhibitor1 (JAKi) was purchased from Calbiochem (420,099). The Human Phospho-RTK Array Kit was purchased from R&D Systems (Proteome Profiler Array, ARY00B). The antibodies used in this study are listed in the Suppl. Materials and Methods.

**Single cell gene expression analysis.** Single cell RNAseq data (5902 cells sequenced from 18 HPV-negative HNSCC) were retrieved from Puram et al. (2017) [15] (dataset GSE103322).

**Gene ontology (GO) analysis**. We used the PANTHER classification system (http://pantherdb.org/) to perform a statistical overrepresentation test. The enrichment of Gene Ontology (GO) terms in our gene set was compared to the whole *Homo Sapiens* genome (GO biological process complete), with a False Discovery Rate (FDR) correction [16].

**QPCR.** Total RNA was extracted and reverse-transcribed using High Capacity cDNA Reverse Transcription kit and random hexamers (Applied Biosystems). Amplification was performed with the TaqMan Universal PCR master Mix on an ABI 7900HT Sequence Detection System (Applied Biosystems). Primers and probe sets for PD-L1 and Glyceraldehyde-Phosphate Dehydrogenase (GAPDH) are described in the Suppl. Material and Methods.

**Immunoblotting.** Complete cell extracts were transferred to nitrocellulose membranes using standard procedures as previously described [17]. The ECL reaction was used to reveal protein. Signal quantifications were performed using ImageJ (https://imagej.nih.gov/ij/download.html).

**Immunofluorescence.** Immunofluorescence labeling of PD-L1 was performed on paraformaldehyde-fixed cells, according to standard procedures [17]. A detailed protocol is given in Suppl. Materials & Methods.

**Statistical analyses.** Analyses were done with R version 4.0.3 (https://www.r-project.org) using packages “Hmisc” and “venneuler”. Student's t-test and ANOVA were used as indicated (GraphPad Prism). The Spearman test was used for gene correlation analyses. *p*<0.05 was used as the threshold of significance. False discovery rate (FDR) correction was applied as indicated using the Bonferroni method.

## Results

### 5-FU upregulates PD-L1 expression in HNSCC cells

**Fig. 1:**
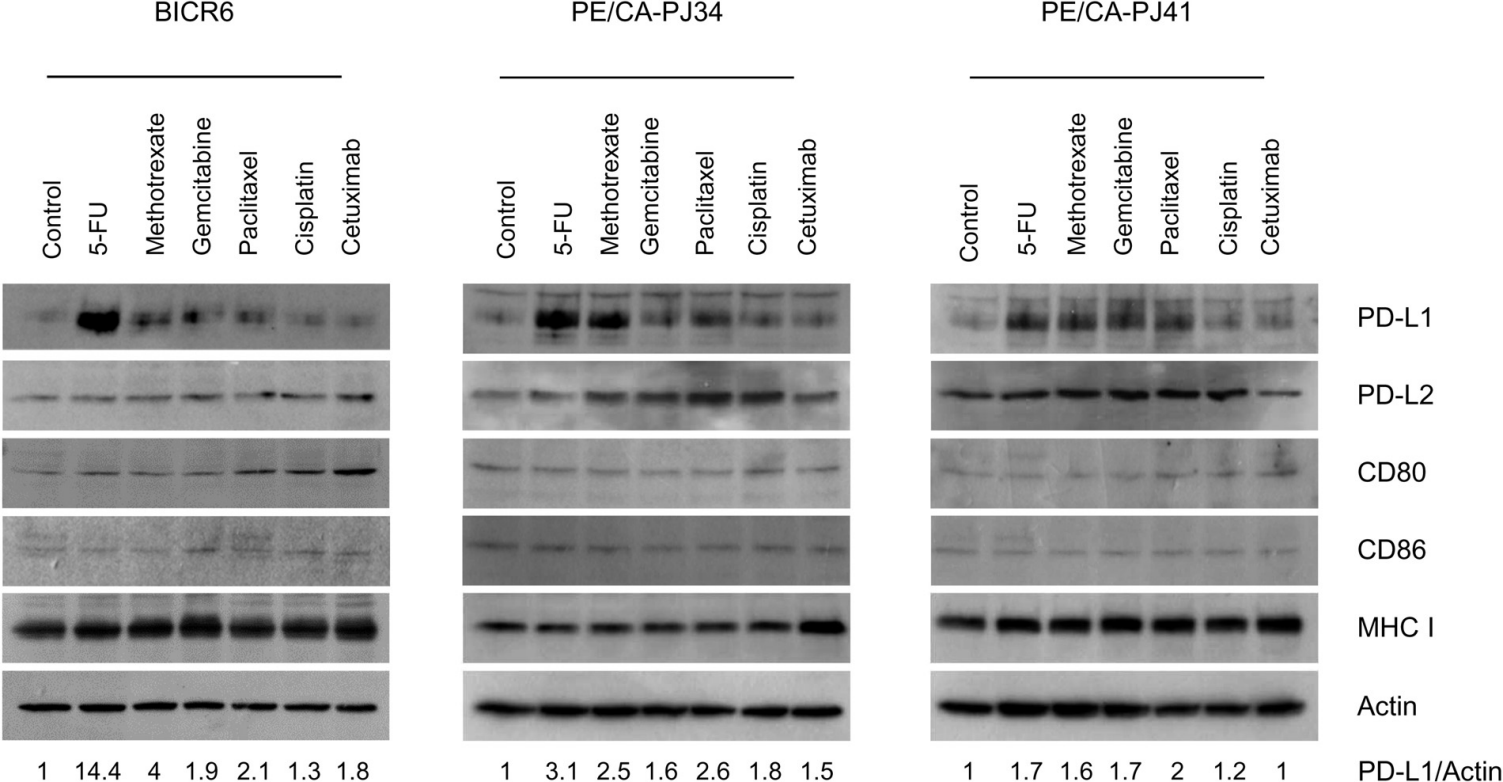
Immunoblot analysis of the expression of the main immune checkpoint molecules in HNSCC cells exposed to chemotherapeutic agents. The cell lines BICR6, PE/CA-PJ34 and PE/CA-PJ41 were exposed to 5-FU, methotrexate, gemcitabine, paclitaxel, cisplatin at IC50 concentrations for 48 h. Cetuximab was applied at a concentration of 50 µg/mL. Expression analysis of the indicated molecules was performed by immunoblotting as indicated. Actin immunolabelling is given as loading control. The indicated values are normalized densitometric analyses of the PD-L1/Actin ratio, taking control condition as 1.

In order to examine the effect of chemotherapeutic agents on HNSCC cells, we used a panel of three HNSCC cell lines (BICR6, PE/CA-PJ34 and PE/CA-PJ41) that were exposed to *n* = 8 chemotherapeutic agents (Fig. 1). All chemotherapeutic agents were applied at concentrations corresponding to their IC_50_, *i.e.* in conditions of comparable efficacy, for 48 h (Suppl. Table 1). Cetuximab was applied at a concentration of 50 µg/mL and was found to block EGFR phosphorylation without significant inhibitory effect on the growth of HNSCC cells *in vitro* (data not shown). We then analyzed the protein expression of PD-L1 as well as PD-L2, CD80, CD86, and MHC class I molecules by immunoblotting (Fig. 1). We observed that 5-FU increased PD-L1 expression in all HNSCC cell lines (fold induction of 14.4, 3.1 and 1.7 compared to control for BICR6, PE/CA-PJ34 and PE/CA-PJ41, respectively) (Suppl. Fig. 1). No effect of 5-FU was detected on the expression of PD-L2, CD80, CD86 and the MHC class I molecules (Fig. 1). In two out of three cell lines, 5-FU seemed to be the chemotherapeutic agent that upregulated PD-L1 expression the most.

### PD-L1 mRNA regulation in single cells from HPV-negative HNSCC tumors

**Fig. 2:**
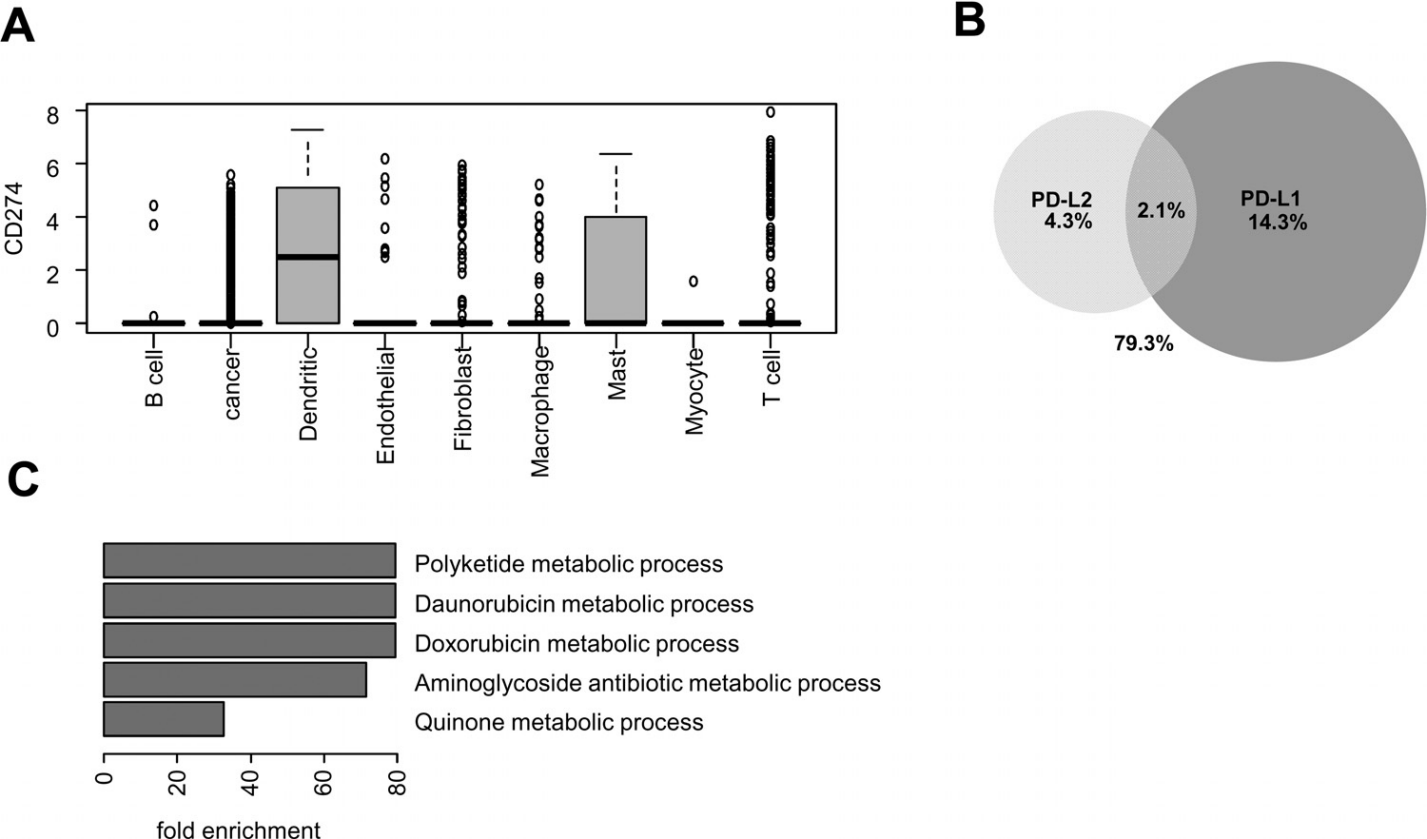
CD274/PD-L1 expression in single cell RNA-seq data retrieved from HPV-negative HNSCC. **A.** Analysis of *CD274*/PD-L1 mRNA levels in single cell RNA-seq data by Puram et al. [15]. *n* = 2215 cancer cells from 18 tumors. **B.** A Venn diagram of the percentage of HNSCC cells expressing PD-L1 and PD-L2 mRNA, and the overlap. **C.** A bar graph showing the Gene Ontology (GO) terms that were statistically overrepresented in the top 150 genes that are significantly correlated with CD274/PD-L1 mRNA in cancer cells, using PANTHER GO overrepresentation test. The figure shows the fold enrichment of each GO term compared to what would be statistically expected.

Single cell RNAseq data from Puram et al. [15] were retrieved in order to examine PD-L1 / PD-L2 mRNA regulation in HNSCC. High levels of PD-L1 mRNA were detected in dendritic cells, mast cells and to a lower extent in HNSCC cells (Fig. 2A). Indeed, 16.4% of tumor cells expressed PD-L1 mRNA (Fig. 2B). PD-L2 was found to be expressed in a smaller fraction of the cancer cells (6.4%) that only minimally overlapped with the population of PD-L1 expressing cells (2.1%) (Fig. 2B). There was no correlation between PD-L1 and PD-L2 mRNA levels in cancer cells (Pearson *r* = 0.05). We identified the genes whose expression was significantly correlated with PD-L1 or PD-L2 mRNA in cancer cells from single cell RNAseq data (Supp. Table 2 and 3, respectively), and compared them with the genes that correlated with PD-L1 mRNA in tumor infiltrating immune cells (Suppl. Table 4). A number of genes were significantly co-expressed with PD-L1, but not with PD-L2 in HNSCC cells (Suppl. Table 2 and 3). These non-overlapping gene contexts suggest the existence of specific regulation of PD-L1 mRNA in HNSCC cells. A statistical overrepresentation test of Gene Ontology terms was performed on the panel of genes found to be correlated with PD-L1 in HNSCC cells, and suggested a link between PD-L1 expression and xenobiotic/chemotherapeutic metabolism (Fig. 1C).

### PD-L1 overexpression induced by 5-FU is related to its genotoxic effect in HNSCC cells

**Fig. 3:**
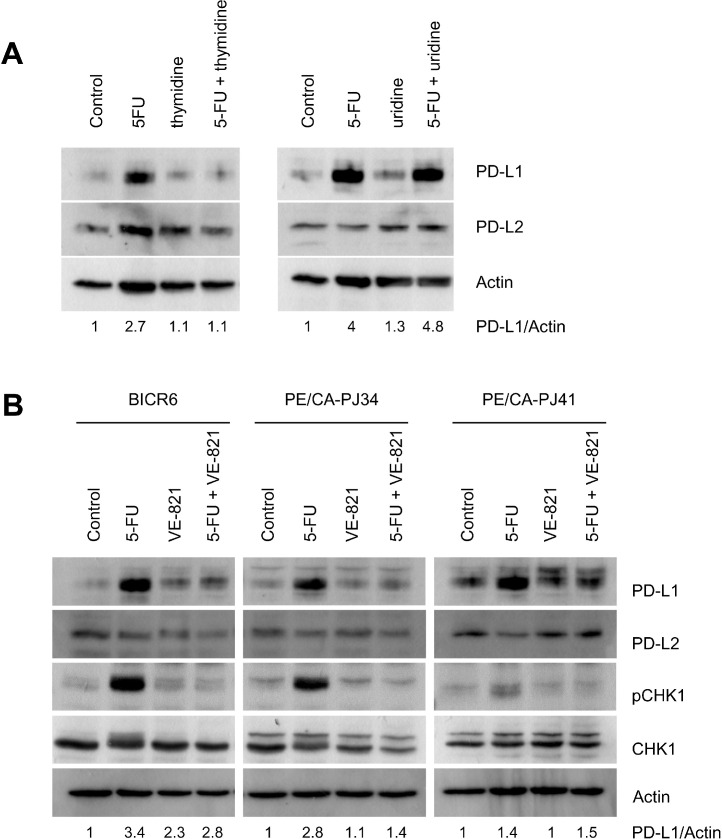
PD-L1 induction in HNSCC cells is related to the genotoxic effect of 5-FU. **A.** Thymidine (20 µM) and uridine (20 µM) were applied extracellularly on BICR6 cells ± 5-FU (IC50) for 48 h. **B.** The chemical inhibitor VE-821 was applied at a final concentration of 10 µM and the cellular extracts were analyzed by immunoblotting with the indicated antibodies.

5-FU can incorporate into RNA or block TS and prevent the synthesis of thymidine. We examined the role played by these two mechanisms by analyzing PD-L1 expression in cells cultivated with extracellularly supplied uridine or thymidine (both at a concentration of 20 µM) (Fig. 3A). We found that extracellular thymidine, but not uridine, was able to revert PD-L1 induction by 5-FU (Fig. 3A, Suppl. Fig. 2A). We next envisioned the possibility that DNA damage response might play a role in PD-L1 induction (Fig. 3B). We used the chemical inhibitor VE-821, directed against the key kinases of the DNA Damage Response ATM/ATR, as previously reported by Ito et al. [9]. We verified that VE-821 prevented Chk1 phosphorylation on Ser345 (a site targeted by activated ATM/ATR) at a concentration of 10 µM (Fig. 3B). Importantly, DNA Damage Response inhibition with VE-821 partially prevented the induction of PD-L1 by 5-FU in the three HNSCC cell lines examined in this study (Fig. 3B, Suppl. Fig. 2B). We concluded that PD-L1 induction by 5-FU was related to its genotoxic effect in HNSCC cells.

### 5-FU and IFN-γ interact to regulate PD-L1 mRNA levels in HNSCC cells

**Fig. 4:**
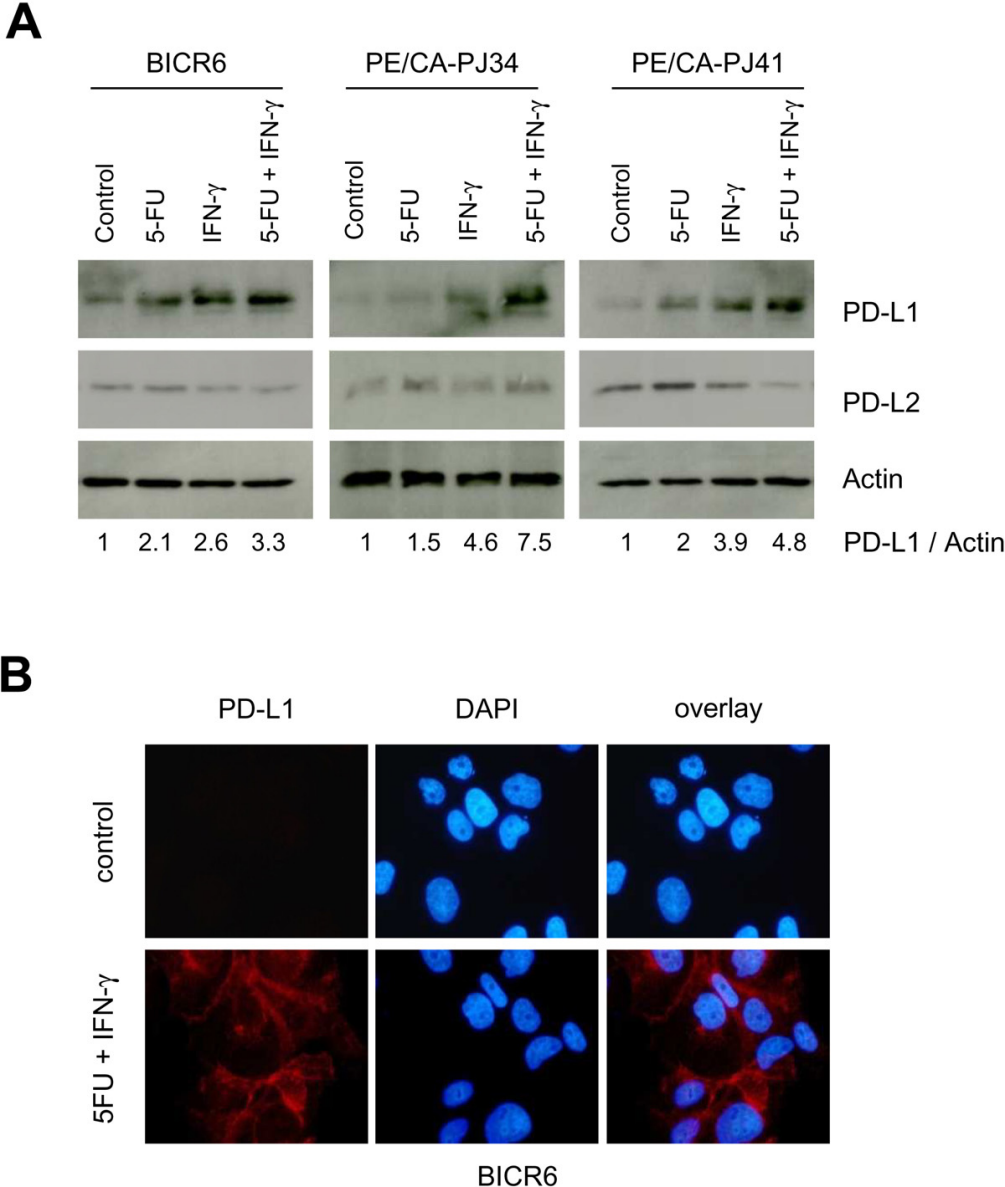
Expression analysis of PD-L1 upon co-exposure of HNSCC cells to 5-FU and IFN-γ. **A.** BICR6, PE/CA-PJ34 and PE/CA-PJ41 cells were exposed to 5-FU and IFN-γ (10 ng/ml) for 24 h, as indicated. **B.** Immunofluorescence analysis of PD-L1 on BICR6 cells exposed to 5-FU + IFN-γ for 24 h (Red fluorescence: PD-L1, blue fluorescence: DAPI).

Interferon γ (IFN-γ) is the canonical inducer of PD-L1 in cancer cells [17]. Compared to various other cytokines or growth factors (IL-6: 10 ng/ml, IL-1β: 1 ng/ml, TNF-α: 25 ng/ml, TGF-β: 5 ng/ml and Nerve Growth Factor, NGF: 40 ng/ml), only IFN-γ (10 ng/ml) robustly induced PD-L1 expression in our experimental conditions (Suppl. Fig. 3). We examined the interaction between 5-FU and IFN-γ in the regulation of PD-L1 (Fig. 4). The co-application of 5-FU with IFN-γ led to an additive induction of PD-L1 in BICR6 and PE/CA-PJ41 cells, and a possible synergy was observed in PE/CA-PJ34 cells (Fig. 4A). A densitometric analysis of the PD-L1/Actin ratio indicated that 5-FU alone induced PD-L1 protein expression (fold induction of 2.1, 1.5 and 2.0 for BICR6, PE/CA-PJ34 and PE/CA-PJ41, respectively), but this induction was greater when the cells were simultaneously exposed to 5-FU and IFN-γ (Fold induction=3.3, 7.5 and 4.8 for BICR6, PE/CA-PJ34 and PE/CA-PJ41, respectively) (Suppl. Fig. 4). Immunofluorescent microscopy detected a clear and homogeneous PD-L1 signal on the surface of cultured BICR6 cells, confirming and extending our observations made by immunoblotting, suggesting that PD-L1 is functional in HNSCC cells exposed to 5-FU + IFN-γ (Fig. 4B).

**Fig. 5:**
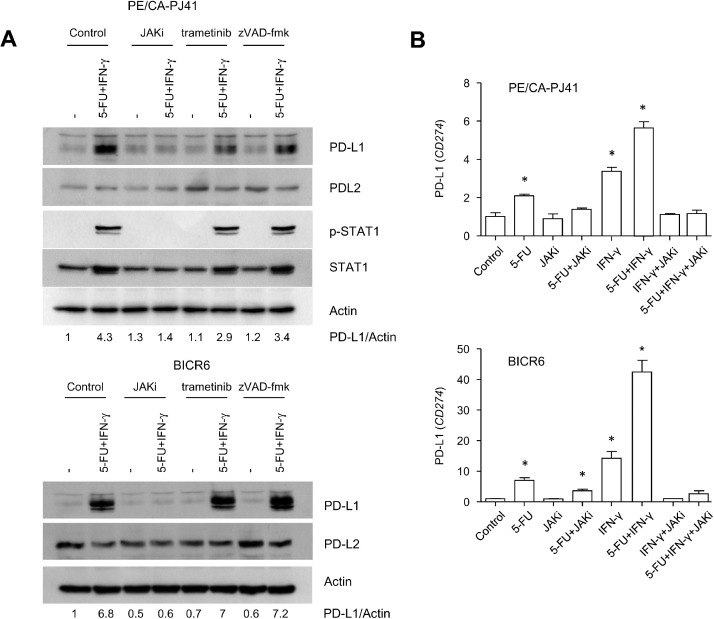
An immunoblot and QPCR analysis of CD274/PD-L1 mRNA expression in HNSCC cells exposed to 5-FU and IFN-γ **A.** BICR6 and PE/CA-PJ41 cells were exposed to 5-FU (IC50) and IFN-γ (10 ng/ml) for 24 h. Cells were preincubated with JAK inhibitor-1 (1 µM), trametinib (1 µM) or zVAD-fmk (50 µM) for 1 h as indicated. Complete cellular extracts were used in order to perform the indicated analyses. Note that STAT1 could not be detected in BICR6 cells. **B.** A QPCR analysis of *CD274*/PD-L1 mRNA expression in PE/CA-PJ41 and BICR6 cells exposed to 5-FU, IFN-γ and JAK inhibitor-1 (1 µM). * *p*<0.05 with Student's t-test compared to control.

We further examined the regulation of PD-L1/*CD274* in BICR6 and PE/CA-PJ41 cells, by performing immunoblot and QPCR analysis (Fig. 5A,B). In order to examine the interaction observed between 5-FU and IFN-γ, we used a broad-spectrum chemical inhibitor of JAK signaling, JAK inhibitor-1 (JAKi) that prevents STAT1 phosphorylation on tyrosine 701 (Fig. 5A). At this concentration, JAKi reduced the clonogenic growth of BICR6 and PE/CA-PJ41 cells, even though its effect was not additive with that of 5-FU (Suppl. Fig. 5). Importantly, JAKi abrogated PD-L1 protein expression in HNSCC cells (Fig. 5A, Suppl. Fig. 6). Meanwhile, we found no effect of trametinib, applied in conditions that radically prevented MEK1/2 phosphorylation (data not shown). The chemical blocker of caspases zVAD-fmk, applied at a concentration of 50 µM blocking apoptosis [19], also had no effect on PD-L1 induction by 5-FU + IFN-γ (Fig. 5A). Similar results were obtained by QPCR (Fig. 5B). While 5-FU and IFN-γ applied as single agents only modestly increased *CD274* mRNA levels, an additive effect was observed upon the co-administration of the two molecules (Fig. 5B). The JAKi abrogated the increased levels of *CD274* mRNA induced by 5-FU + IFN-γ (Fig. 5B). We concluded that 5-FU and IFN-γ converge on JAK signaling to regulate CD274/PD-L1 expression at the transcriptional level.

### EGFR as an actionable target to prevent PD-L1 induction by 5-FU in HNSCC cells

**Fig. 6:**
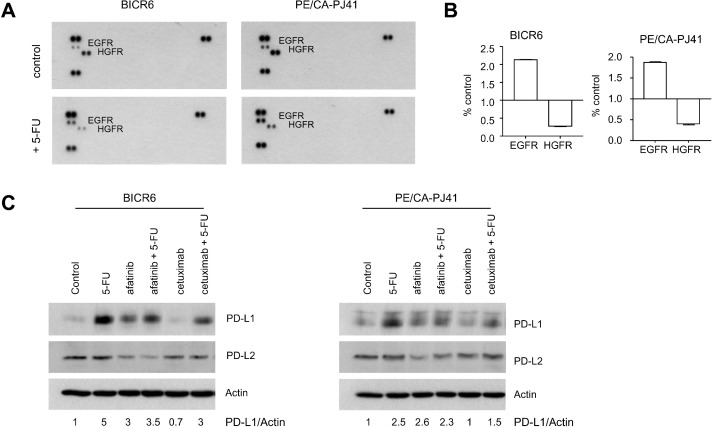
EGFR as an actionable target for preventing PD-L1 induction by 5-FU in HNSCC cells **A.** A phospho-RTK array preformed using cellular extracts prepared from BICR6 and PE/CA-PJ41 cells, either in control conditions or after exposure to 5-FU (IC50) for 48 h **B.** Quantification of EGFR and HGFR phosphorylation after 5-FU treatment, taking control conditions as reference for each cell line. **C.** Immunoblot analysis of cellular extracts prepared from BICR6 and PE/CA-PJ41 cells exposed to 5-FU (IC50), afatinib (5 µM) and cetuximab (50 µg/ml) for 48 h, as indicated. The indicated values are normalized densitometric analyses of the PD-L1/Actin ratio, taking control condition as 1.

A previous study reported the existence of PD-L1 regulation downstream of the Receptor Tyrosine Kinase (RTK) EGFR [20]. We therefore explored the phosphorylation status of 49 RTK in HNSCC cells. A phospho-RTK array indicated that BICR6 and PE/CA-PJ41 mainly expressed phosphorylated forms of EGFR and HGFR (Hepatocyte Growth Factor Receptor), while the other RTK were detectable at considerably lower levels (Fig. 6A). Importantly, HNSCC cells exposed to 5-FU at IC50 concentration for 48 h had increased phosphorylation levels of EGFR, with simultaneously reduced HGFR phosphorylation levels (Fig. 6A,B). The increase in EGFR phosphorylation was quantified to be 5-fold in BICR6 and 2.5-fold in PE/CA-PJ41 exposed to 5-FU for 48 h (Fig. 6B, Suppl. Fig. 7). We used two different approaches that inhibit EGFR kinase activity to examine the role of EGFR in this setting: the application of cetuximab (50 µg/ml) or the chemical inhibitor afatinib (5 µM). While a slight upregulation of PD-L1 was induced by afatinib as single agent in basal conditions, both cetuximab and afatinib inhibited PD-L1 overexpression induced by 5-FU to an extent of around 50% in BICR6 and PE/CA-PJ41 (Fig. 6C). We concluded that EGFR targeting partially prevents PD-L1 expression in HNSCC cells exposed to 5-FU.

## Discussion

In the present study, we examined the effects of various chemotherapeutic agents on HNSCC cells and observed that 5-FU robustly increased PD-L1 expression. This induction was observed in three independent genomic contexts, suggesting its potential broad relevance. A clear interaction (either additive or synergistic, depending on the cellular context) was observed between 5-FU and IFN-γ. PD-L1 was homogeneously distributed on the surface of the whole population of HNSCC cells, suggesting that it was not accounted for by a minor subpopulation of cancer cells and that PD-L1 is functional in this context. The effect of 5-FU on PD-L1 expression appeared to be related to its genotoxic effect and was prevented by the extracellular application of thymidine or by the DNA damage Response inhibitor VE-821, directed against the kinases ATM/ATR. Finally, we found that EGFR inhibition partially prevented PD-L1 induction by 5-FU.

Recent studies point to the regulation of PD-L1 by oncogenic, inflammatory and hypoxic signaling in cancer cells [21]. A few studies have addressed the impact of therapeutic agents used against HNSCC on the expression of PD-L1 [13,14,22]. To the best of our knowledge, our report shows for the first time that 5-FU is an inducer of PD-L1 expression in HNSCC cells. Recent studies performed in various types of primary tumors point to similar effects of 5-FU in digestive cancers [23,24]. Another antimetabolite with a related mode of action, pemetrexed, was also recently found to induce PD-L1 in Non-Small Cell Lung Cancer cells [25]. Our observation that the regulation of PD-L1 in cancer cells does not entirely overlap with that of immune cells confirms the data of Chen et al.*,* although the corresponding study did not examine the effects of xenobiotics/chemotherapeutics [18]. We verified that the induction of PD-L1 was not accounted for either by cell senescence or the loss of cancer cell viability in this context (data not shown). Importantly, it was possible to abolish the effect of the dual exposure of HNSCC cells to 5-FU and IFN-γ with a chemical inhibitor active against JAK. JAK signaling in cancer cells might therefore constitute a point of convergence and a key control of the transcriptional induction of PD-L1 in HNSCC cells exposed to chemotherapies. Non-selectively targeting JAK signaling in cancer patients would target tumor infiltrating immune cells in addition to cancer cells [26], and it is therefore difficult to anticipate the therapeutic interest of this strategy.

We examined the possibility of targeting the induction of PD-L1 using already approved anti-cancer drugs. We observed that EGFR signaling is induced by 5-FU and that its inhibition by cetuximab prevents PD-L1 upregulation. Previous studies found that oncogenic signaling downstream of the growth factor receptors (EGFR and HGFR) positively regulates PD-L1 expression in HNSCC cells [20,27,28]. However, the corresponding studies did not address the effects of chemotherapeutics. Importantly, a previous study even reported a striking convergence of EGFR and IFN-γ signaling in the regulation of JAK-STAT and PD-L1 expression in HNSCC [28]. In esophageal cancer cells exposed to a conventional chemotherapy regimen, an induction of PD-L1 was also observed that was prevented by blocking EGFR [29]. While this observation is reminiscent of our findings, the contribution of 5-FU was not directly addressed in this study [29]. Importantly, the fact that cetuximab counteracts PD-L1 induction suggests that autocrine/paracrine activation of EGFR occurs in this context. Further studies addressing the composition of the cancer cell secretome and the regulation of JAK-STAT axis are warranted. Another key question worth addressing is the role of the DNA damage response in this context [30].

Activation of an adaptive immune response directed against cancer cells is emerging as a mechanism that contributes to the efficacy of chemotherapeutic protocols [10,11]. The present study did not include an *in vivo* experimental part with an immunocompetent animal model. However, based on previous studies that examined the role of PD-L1 in cancer cells in human HNSCC samples [6,7], we postulate that the induction of PD-L1 by 5-FU might limit the adaptive immune response against cancer in patients receiving radio(chemo)therapy. Our study therefore raises interesting possibilities regarding the use of ICB in HNSCC. Chemotherapeutic agents remain an important therapeutic modality and are often combined with ICB against R/M HNSCC [31]. 40% of HNSCC show an enriched inflammatory response with active interferon-γ signaling [32]. The tumor microenvironment is emerging as a key player in the regulation of the adaptive immune response against solid tumors [33]. Our observations further suggest the importance of the tumor microenvironment in the therapeutic context. Our study also provides a biological rationale for targeting PD1 in association with chemotherapeutic regimen containing 5-FU, especially when a tumor has a dense T cell infiltrate/high local production of IFN-γ. Clinical studies are needed to examine this possibility.

## Author contributions statement

**C.L.** Conceptualization, Investigation, Writing: Review & Editing; **M.L.** Writing: Review & Editing; **F.R.** Investigation, Writing: Review & Editing; **C.L.**: Investigation; **C.G.**: Investigation; **B.C.**: Supervision, Funding acquisition, Writing: Review & Editing; **A.G.**: Conceptualization, Funding acquisition, Project administration, Writing: Original Draft; **Z.S.**: Conceptualization, Project administration, Writing: Original Draft.

## Declaration of Competing Interest

The authors declare that they have no conflict of interest to disclose for this work.

## Appendix Supplementary materials

Image, application 1

Image, application 2
