# Supplementary material for: DNA damage response- and JAK-dependent regulation of PD-L1 expression in head and neck squamous cell carcinoma (HNSCC) cells exposed to 5-fluorouracil (5-FU)
Source: Transl Oncol. 2021 May 2;14(8):101110. doi: 10.1016/j.tranon.2021.101110 (PMC8111315; doi:10.1016/j.tranon.2021.101110)
Supplement: Supplementary file 1 [file mmc1.docx]

**Supplementary Materials and Methods**

**DNA damage response- and JAK-dependent regulation of PD-L1 expression in Head and Neck Squamous Cell Carcinoma (HNSCC) cells exposed to 5-Fluorouracil (5-FU)**

Claire Lailler *et al.*

**Cell cultures**

All cell lines used in this study are HPV^-ve^ HNSCC. The cell line BICR6 derives from a hypopharynx tumor and bears a homozygous mutant version of *TP53* Gln192Ter ([cellosaurus CVCL_2314](https://web.expasy.org/cellosaurus/CVCL_2314)). PE/CA-PJ34 is homozygous for *TP53* Ala159Val [(cellosaurus CVCL_2679](https://web.expasy.org/cellosaurus/CVCL_2679)). PE/CA-PJ41 derives from an oral cavity tumor and is homozygous for TP53 920-2A>T (splice acceptor mutation) ([cellosaurus CVCL_2680](https://web.expasy.org/cellosaurus/CVCL_2680)). All Cell lines were purchased from ECACC (European Collection of Authenticated Cell Cultures) and they were authenticated by Short tandem repeat (STR) profiling (ATCC) less than one year prior to the experiments.

**Reagents**

All cytokines except for IL-1β were purchased from R&D System Biotechne: IL-6 (reference 206-IL), IFN-γ (285-IF), TNF-α (210-TA), TGF-β1 (240-B) and NGF (256-GF). IL-1β was purchased from PeproTech (reference 200-01B).

**Antibodies**

Rabbit anti-phospho-STAT1 (Y701) and anti-phospho-Chk1 (S345) were purchased from Cell Signalling (references 7649 and 2348, respectively). Mouse anti-Actin was purchased from Sigma-Aldrich (A5441). All the other antibodies were purchased from Abcam: anti-CD80 (ab134120), anti-CD86 (ab269587), anti-MHC I (ab70328), anti-PD-L1 (ab213524), anti-PD-L2 (ab187662), anti-STAT1 (ab109320), anti-Chk1 (ab32531). The secondary antibodies used for immunoblotting were purchased from GE Healthcare: anti-rabbit IgG HRP-linked (LNA934V/AH) and anti-mouse IgG HRP linked (LNA931V/AH).

**Immunofluorescence labelling of PD-L1**

Cells grown on glass coverslips were fixed with 3.7% paraformaldehyde, permeabilized with 0.01% Triton X100, and stained with relevant antibodies. Nuclei were stained with diamidino-phenylindole (DAPI). Coverslips were mounted in Mowiol (Calbiochem) and observed with a Nikon Eclipse TE2000U microscope equipped with a plan APO VC 60X / 1.40 objective under oil immersion. Images were processed and superposed with the Photoshop software (Adobe).

**Probe for Taqman Gene Expression Assay**

*CD274* expression was analyzed with a FAM-MGB probe purchased from Thermofisher Scientific (reference Hs00204257_m1).
