# Supplementary material for: DNA damage response- and JAK-dependent regulation of PD-L1 expression in head and neck squamous cell carcinoma (HNSCC) cells exposed to 5-fluorouracil (5-FU)
Source: Transl Oncol. 2021 May 2;14(8):101110. doi: 10.1016/j.tranon.2021.101110 (PMC8111315; doi:10.1016/j.tranon.2021.101110)
Supplement: Supplementary file 2 [file mmc2.docx]

**Supplementary Tables and Figures**

**DNA damage response- and JAK-dependent regulation of PD-L1 expression in Head and Neck Squamous Cell Carcinoma (HNSCC) cells exposed to 5-Fluorouracil (5-FU)**

Claire Lailler *et al.*

**Supp. Table 1:** IC50 measured for chemotherapeutic agents in HNSCC cell lines

**Supp. Table 2:** Top 25 genes positively correlated with PD-L1/CD274 in cancer cells

**Supp. Table 3:** Top 25 genes positively correlated with PD-L2 in cancer cells

**Supp. Table 4:** Top 25 genes positively correlated with PD-L1 in tumor infiltrating immune cells

**Supp. Fig. 1: Quantification of PD-L1/Actin ratio in HNSCC cells exposed to different chemotherapeutic agents.**

**Supp. Fig. 2: Quantification of PD-L1/Actin ratio in HNSCC cells exposed to 5-FU and thymidine/uridine (panel A) or the checkpoint kinase inhibitor VE-821 (panel B).**

**Supp. Fig. 3:** Immunoblots of PD-L1 in BICR6 cells exposed to different cytokines.

**Supp. Fig. 4: Quantification of PD-L1/Actin ratio in HNSCC cells exposed to 5-FU and Ifn-γ.**

**Supp. Fig. 5:** Analysis of cell viability in HNSCC cells exposed to 5-FU + JAKi

**Supp. Fig. 6: Quantification of PD-L1/Actin ratio in HNSCC cells exposed to 5-FU+Ifn-γ, and effect of the chemical inhibitors JAKi, trametinib and zVAD-fmk.**

**Supp. Fig. 7: Quantification of PD-L1/Actin ratio in HNSCC cells exposed to 5-FU and afatinib / cetuximab.**

**Supp. Table 1: IC50 measured for chemotherapeutic agents in HNSCC cell lines**

**BICR6 PE/CA-PJ41 PE/CA-PJ34**

**Drugs IC50 (M) Log IC50 IC50 (M) Log IC50 IC50 (M) Log IC50**

doxorubicin 4.11x10^-7^ -6.39 1.53x10^-7^  -6.82 5.08x10^-6^ -5.29

oxaliplatin 3.49x10^-6^ -5.46 6.12x10^-5^ -4.21 4.65x10^-5^ -4.33

5-FU 1.72x10^-6^ -5.77 4.08x10^-6^ -5.39 1.03x10^-5^ -4.99

methotrexate 2.92x10^-8^ -7.53 3.39x10^-8^ -7.47 - -

gemcitabine 1.25x10^-8^ -7.9 6.53x10^-8^ -7.19 1.08x10^-6^ -5.97

paclitaxel 9.69x10^-9^ -8.01 8.01x10^-8^ -7.1 8.66x10^-8^ -7.06

cisplatin 9.45x10^-6^ -5.02 1.07x10^-5^ -4.97 2.67x10^-5^ -4.57

Footnote: The IC50 were determined from cell viability assay results, using log concentration of each drug versus response. A nonlinear fit was determined using Sigmoidal fitting (Graphpad Prism).

**Supp. Table 2: Top 25 genes positively correlated with PD-L1/CD274 in cancer cells**

**Gene Spearman r p (FDR)**

CD274 1.00 0.0000

MEGF10 0.29 0.0000

GSTM4 0.27 0.0000

TMEM116 0.27 0.0000

AKR1C1 0.26 0.0000

MYH11 0.25 0.0000

MAP1B 0.25 0.0000

DLEC1 0.25 0.0000

RAB6B 0.25 0.0000

ALDH1A1 0.25 0.0000

KIAA1549L 0.25 0.0000

UGT1A7 0.25 0.0000

AKR1C2 0.24 0.0000

OLFM1 0.24 0.0000

NTM 0.24 0.0000

MATK 0.24 0.0000

SLC9A3R1 0.24 0.0000

LOC344887 0.24 0.0000

SRXN1 0.24 0.0000

ADH7 0.24 0.0000

TSPAN18 0.23 0.0000

FKBP10 0.23 0.0000

WNT5A 0.23 0.0000

CYP4F3 0.23 0.0000

NTRK2 0.23 0.0000

UGT1A10 0.23 0.0000

Footnote: Single-cell RNA seq data from Puram et al. (2017) was used to identify genes correlated with PD-L1 (CD274) in n=2215 HNSCC cells from 18 patients. The above table shows the top 25 genes out of the 2122 significantly positively correlated genes after FDR correction of the p value. Total number of genes tested = 23686.

**Supp. Table 3: Top 25 genes positively correlated with PD-L2 in cancer cells**

**Gene Spearman r p (FDR)**

PDCD1LG2 1.00 0.0000

ABCC11 0.16 0.0000

HRNR 0.15 0.0000

FCAMR 0.14 0.0000

CLDN8 0.14 0.0000

MAGEB2 0.14 0.0000

KLHL4 0.13 0.0000

KATNB1 0.13 0.0000

ELOVL6 0.13 0.0000

ENTPD3 0.13 0.0000

KIAA1549L 0.13 0.0000

SEPT11 0.13 0.0000

SEPT5 0.13 0.0001

EIF5A 0.12 0.0001

SLC25A1 0.12 0.0001

DPY19L3 0.12 0.0001

G6PD 0.12 0.0001

ADAM23 0.12 0.0001

NPLOC4 0.12 0.0001

IKBKE 0.12 0.0002

MIR4517 0.12 0.0003

LINC00692 0.12 0.0003

FADS2 0.12 0.0004

PRDM16 0.12 0.0004

USB1 0.12 0.0004

Footnote: Single-cell RNA seq data from Puram et al. (2017) was used to identify genes correlated with PD-L2 (PDCD1LG2) in n=2215 HNSCC cells from 18 patients. The above table shows the top 25 genes out of the 166 significantly positively correlated genes after FDR correction of the p value. Total number of genes tested = 23686.

**Supp. Table 4: Top 25 genes positively correlated with PD-L1 in tumor infiltrating immune cells**

**Gene Spearman r p (FDR)**

CD274 1.00 0.0000

FSCN1 0.31 0.0000

TYROBP 0.31 0.0000

TMEM176B 0.30 0.0000

GSN 0.30 0.0000

CD83 0.30 0.0000

TMEM176A 0.30 0.0000

CST3 0.28 0.0000

ALOX5 0.28 0.0000

KIT 0.26 0.0000

AKAP2 0.25 0.0000

NEK6 0.25 0.0000

FCER1G 0.25 0.0000

CLIC2 0.25 0.0000

TPSD1 0.25 0.0000

CAPG 0.25 0.0000

ABP1 0.25 0.0000

SLC18A2 0.25 0.0000

PTGS1 0.25 0.0000

LAD1 0.25 0.0000

HDC 0.25 0.0000

IL1RL1 0.25 0.0000

ANXA3 0.25 0.0000

LIMCH1 0.25 0.0000

SPINT2 0.24 0.0000

RNF130 0.24 0.0000

Footnote: Single-cell RNA seq data from Puram et al. (2017) was used to identify genes correlated with PD-L1 (CD274) in n=1662 tumor infiltrating immune cells (macrophages, mast cells, dendritic cells, B cells, T cells) from 18 patients. The above table shows the top 25 genes out of 760 genes that were significantly positively correlated with PD-L1 after FDR correction of the p value. Total number of genes tested = 23686.


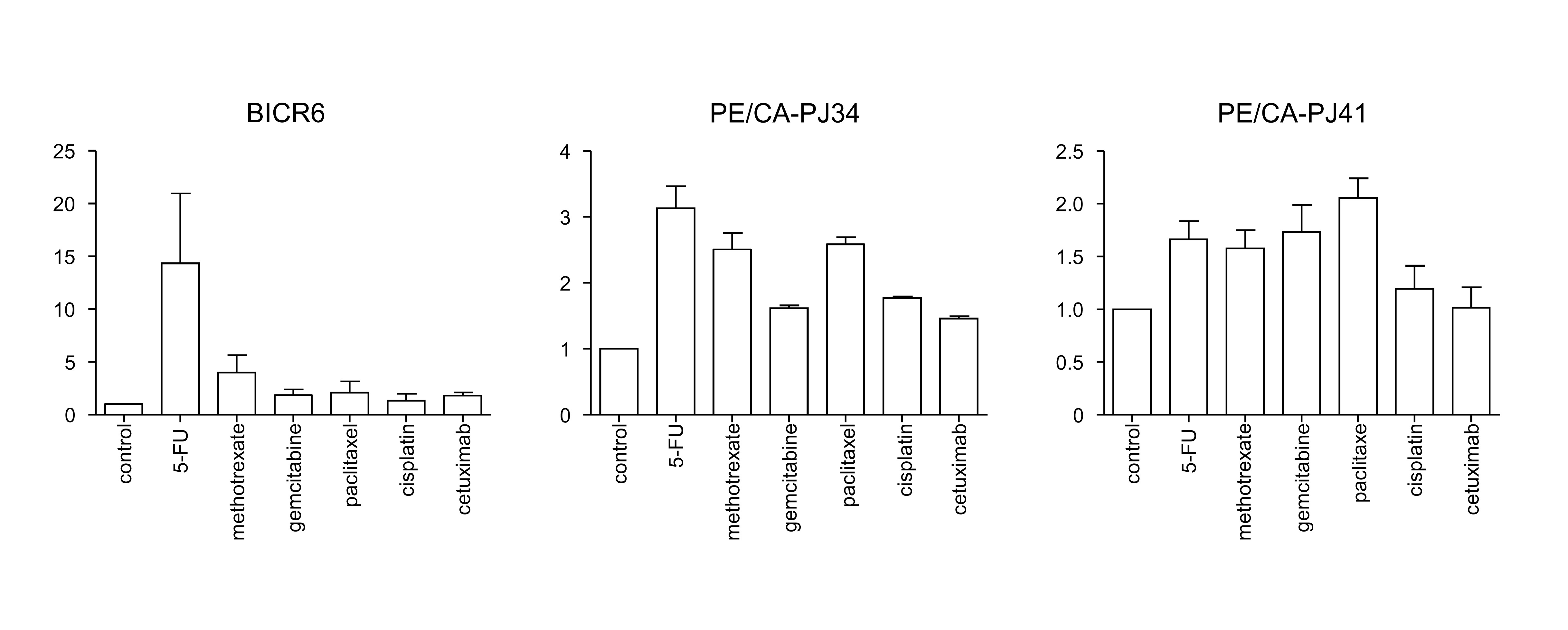


**Supp. Fig. 1: Quantification of PD-L1/Actin ratio in HNSCC cells exposed to different chemotherapeutic agents.** The cell lines BICR6, PE/CA-PJ34 and PE/CA-PJ41 were exposed to 5-FU, methotrexate, gemcitabine, paclitaxel, cisplatin at IC50 concentrations for 48 hours. Cetuximab was applied at a concentration of 50 µg/mL. The indicated values are normalized densitometric analyses of the PD-L1/Actin ratio, taking control condition as 1 (average of n=3, p<0.05 using ANOVA).


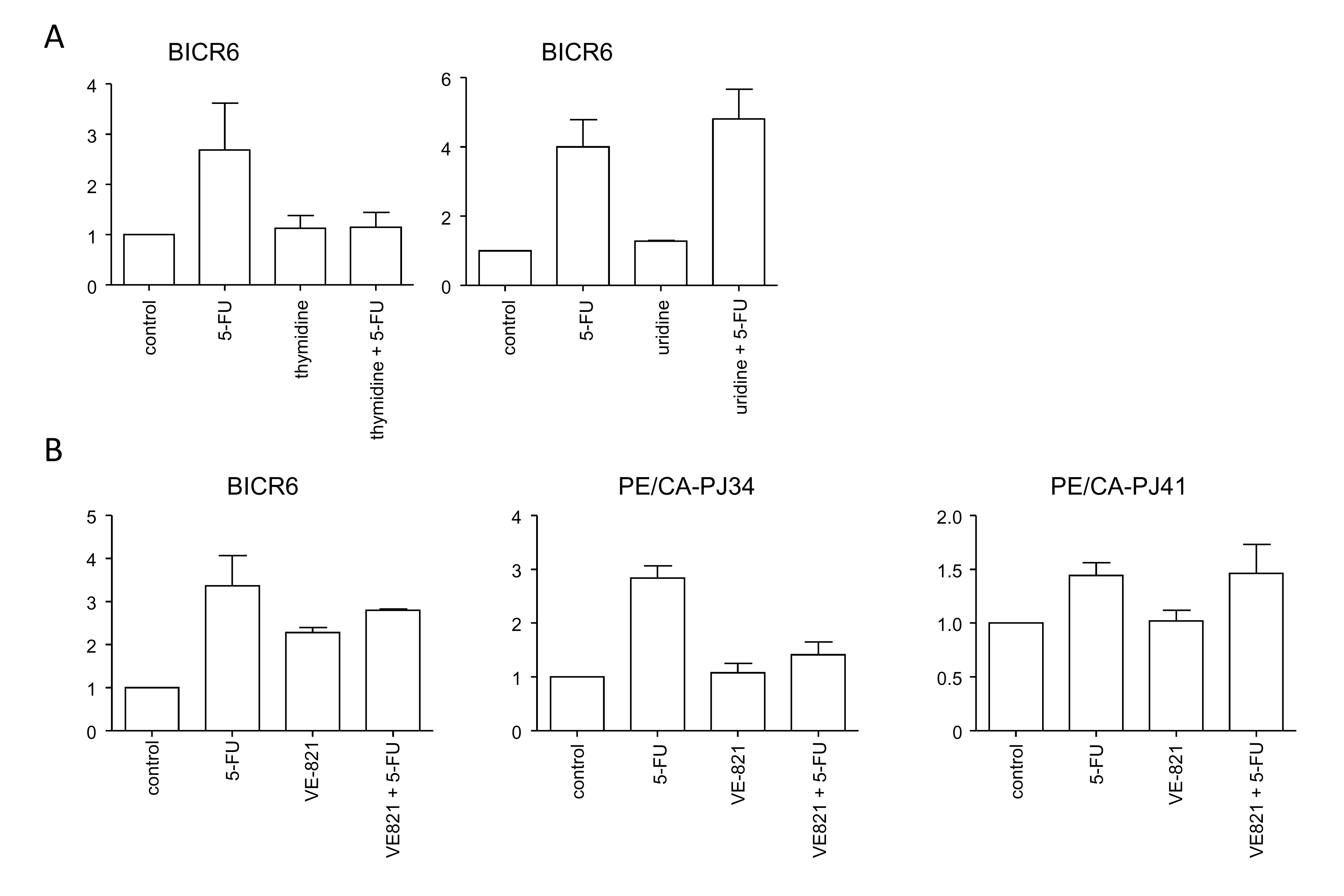


**Supp. Fig. 2: Quantification of PD-L1/Actin ratio in HNSCC cells exposed to 5-FU and thymidine/uridine (panel A) or the checkpoint kinase inhibitor VE-821 (panel B).** The indicated values are normalized densitometric analyses of the PD-L1/Actin ratio, taking control condition as 1 (average of n=3, p<0.05 using ANOVA).

Actin

0

TNF-α

IL-6

IFN-γ

IL-1β

TGF-β1

NGF

PD-L1


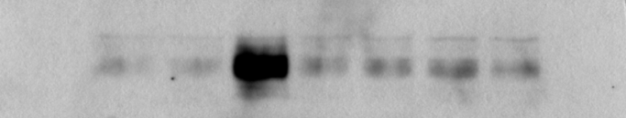

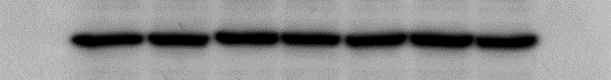


**Supp. Fig 3: Immunoblots of PD-L1 in BICR6 cells exposed to different cytokines.**

The cell line BICR6 was exposed to IL-6 (10 ng/ml), IFN-γ (10 ng/ml), IL-1β (1 ng/ml), TNF-α (25 ng/ml), TGF-β1 (5 ng/ml) or NGF (40 ng/ml) for 48 hours. Actin was used as a loading control.


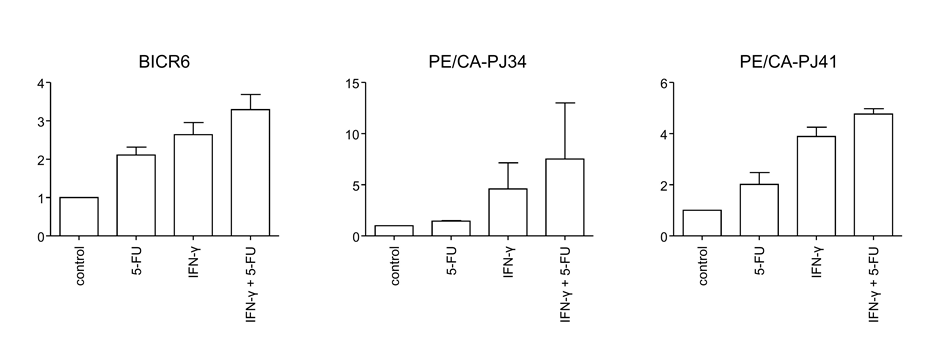


**Supp. Fig. 4: Quantification of PD-L1/Actin ratio in HNSCC cells exposed to 5-FU and Ifn-γ.** The indicated values are normalized densitometric analyses of the PD-L1/Actin ratio, taking control condition as 1 (average of n=3, p<0.05 using ANOVA).


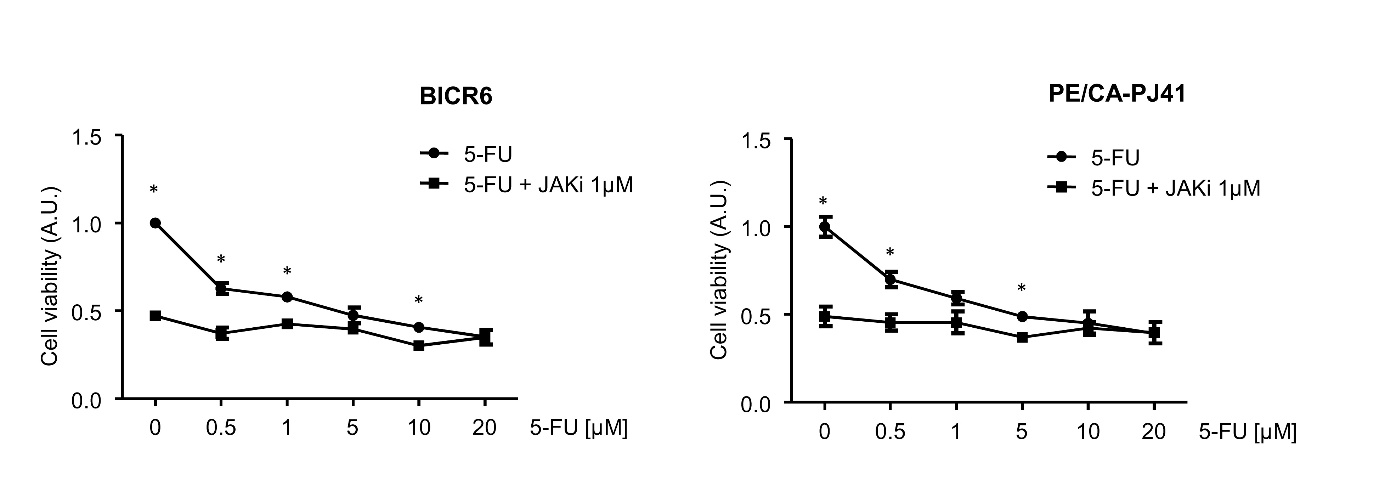


**Suppl. Fig. 5: Analysis of cell viability in HNSCC cells exposed to 5-FU + JAKi**

Cell viability was analyzed after 48h of culture in the indicated conditions (increasing concentrations of 5-FU + 1 µM JAKi). * indicates a significant difference between the condition with/without JAK inhibitor.


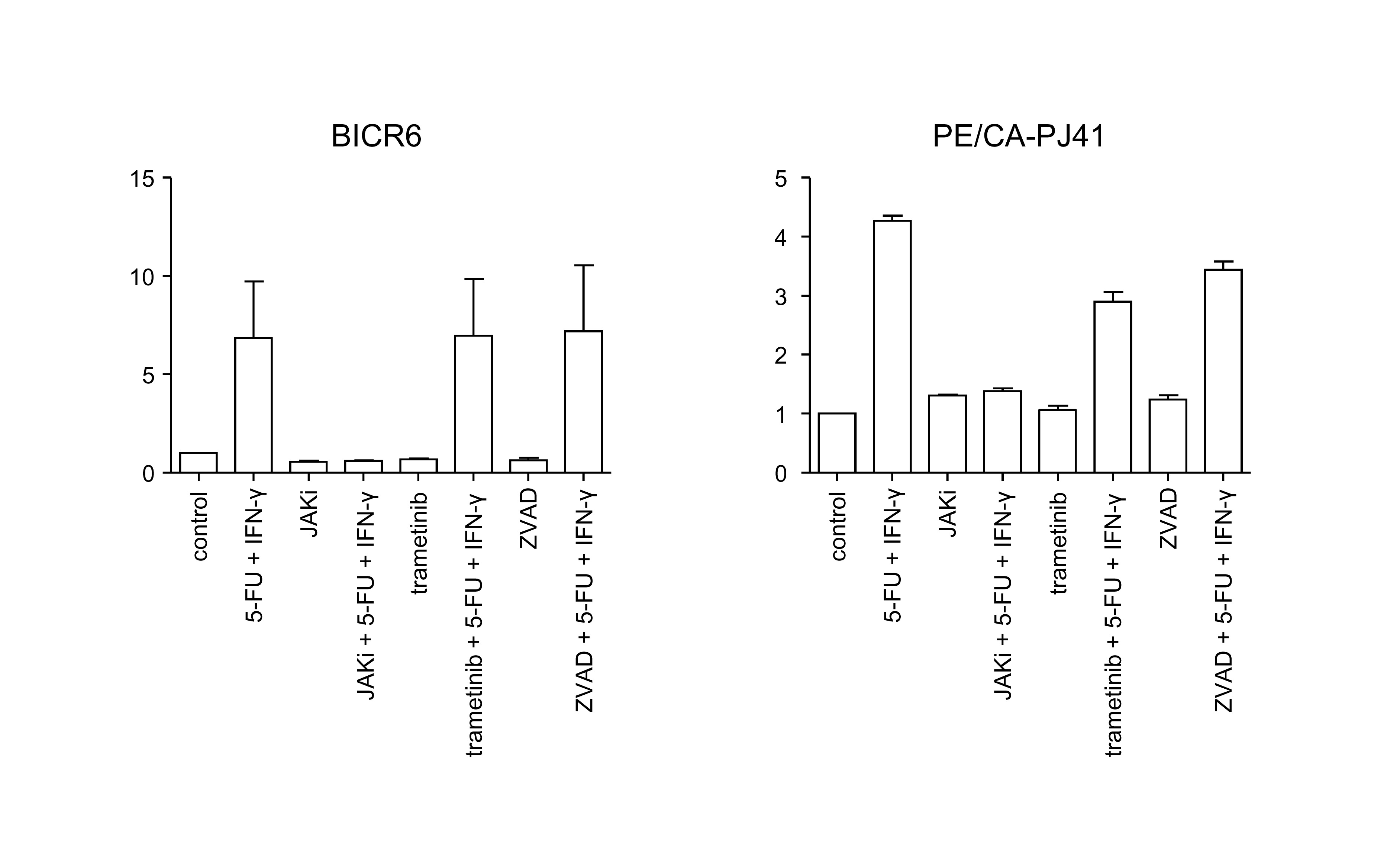


**Supp. Fig. 6: Quantification of PD-L1/Actin ratio in HNSCC cells exposed to 5-FU+IFN-γ, and effect of the chemical inhibitors JAKi, trametinib and zVAD-fmk.** The indicated values are normalized densitometric analyses of the PD-L1/Actin ratio, taking control condition as 1 (average of n=3, p<0.05 using ANOVA).


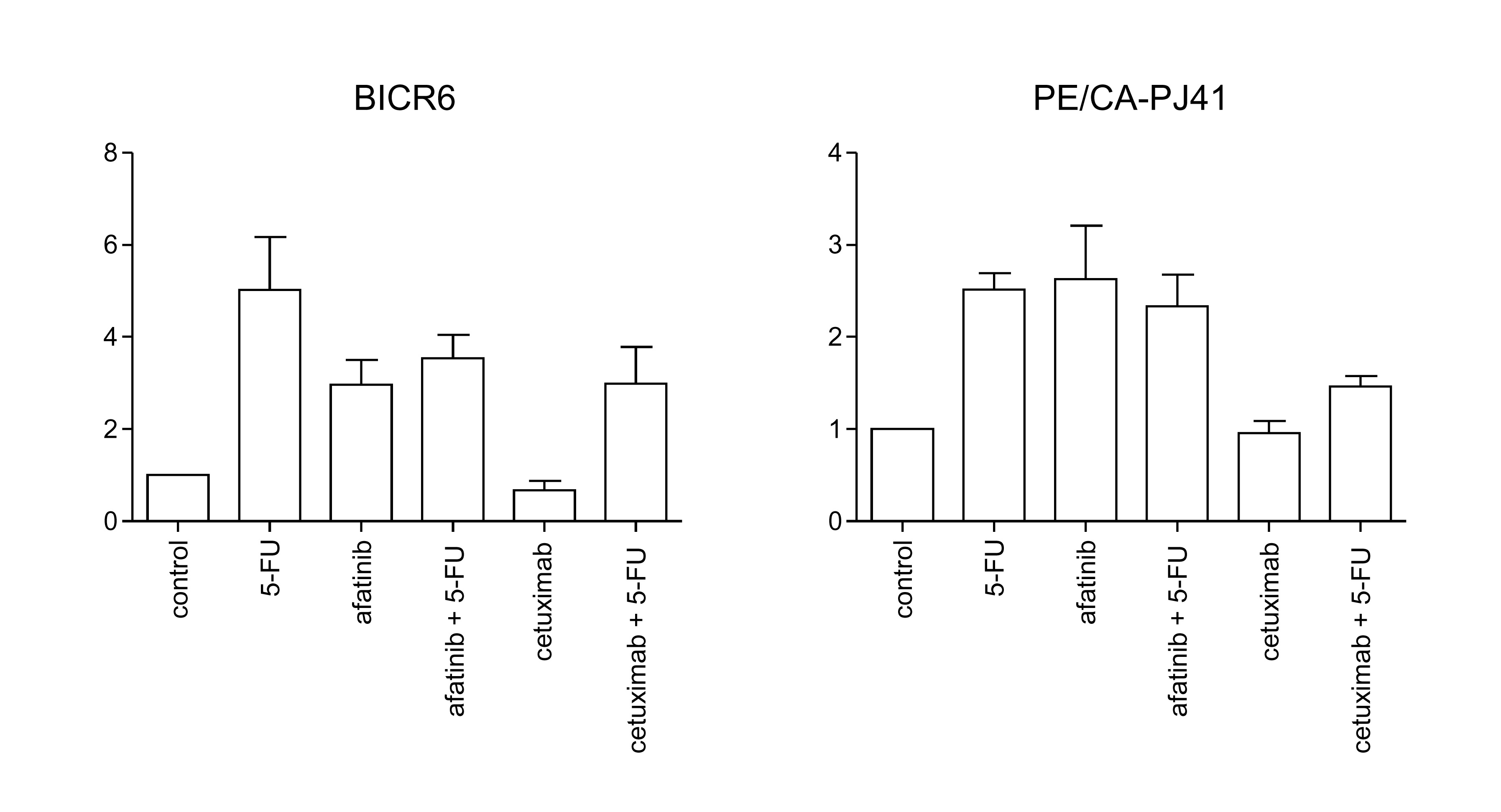


**Supp. Fig. 7: Quantification of PD-L1/Actin ratio in HNSCC cells exposed to 5-FU and afatinib / cetuximab.** The indicated values are normalized densitometric analyses of the PD-L1/Actin ratio, taking control condition as 1 (average of n=3, p<0.05 using ANOVA).
